# Determinants of psychosocial wellbeing within a year-long youth cycling program

**DOI:** 10.3389/fspor.2026.1892082

**Published:** 2026-10-19

**Authors:** Emily M. Adamic, Michael J. Wozniak, Casandra M. Dickerson, Lance C. Miller, Jonah L. Panther, Adam Vanderberg, Jason Whorton, Cian L. Brown

**Affiliations:** 1 Bike Club, Tulsa, OK, United States; 2 Department of Counseling, Leadership, and Research Methods, University of Arkansas, Fayetteville, AR, United States

**Keywords:** cycling, outdoor recreation, psychosocial wellbeing, school-based intervention, youth mental health

## Abstract

**Background:**

School-based cycling programs are increasingly being implemented as accessible means to promote overall wellbeing in youth through structured physical activity. As these programs continue to develop, more rigorous research approaches can be used to better understand how these programs impact participant wellbeing outcomes. Here, we sought to examine within-subject trends and identify multivariate predictors of psychosocial wellbeing among youth participating in “Bike Club”, a year-long after-school cycling program.

**Methods:**

We studied 329 fourth- through eighth-grade students enrolled in Bike Club across 37 schools in Tulsa, Oklahoma. We assessed pre- and post-program psychosocial wellbeing using survey-based scales, and used mixed-effects stepwise linear regression to explore the relationship of individual changes in wellbeing with self-reported physical activity, outdoor time, perceived help with emotions, connection with nature, and bike self-efficacy, along with demographic variables.

**Results:**

Initial paired analysis indicated no significant group-level change in wellbeing between pre- and post-program, despite substantial individual variability (56% of participants reported meaningful changes). Perceived help with emotions and physical activity were most closely associated with wellbeing, followed by modifiable variables like outdoor time and bike self-efficacy and demographic variables like gender, race and ethnicity, and English language learning status. Although the program significantly increased bike self-efficacy and daily outdoor time, it did not appear to significantly relate to the frequency of physical activity or perceived emotional support.

**Conclusion:**

While school-based cycling programs may support positive youth development with the potential to influence multiple correlates of wellbeing, our findings suggest they may not independently produce consistent mental health improvements when relying on incidental activity exposure alone. Perhaps intentional integration of psychosocial or therapeutic components is needed for cycling programs to support improvement in wellbeing, with longitudinal study designs across multiple timepoints and comparison groups to better isolate program effects while integrating more targeted intervention strategies.

## 1 Introduction

School-based cycling interventions are brief, curriculum-based physical education or elective programs designed to teach cycling skills, increase physical activity, and promote healthy lifestyle behaviors. Importantly, such programs are increasingly recognized as feasible interventions that also contribute to adolescent mental health and wellbeing. Across program evaluations, brief and validated mental health screening tools such as the World Health Organization's five-question well-being index (WHO-5) and the Youth Pediatric Symptom Checklist (PSC-17-Y) have demonstrated small but consistent improvements in self-reported wellbeing following participation (1–7). However, these effects may be heterogeneous across individuals, and are influenced by demographic characteristics such as gender, race and ethnicity, and socioeconomic background, as well as modifiable lifestyle factors such as physical activity, sleep, and screen time (4, 5). Collectively, while findings support that school-based cycling programs may offer a feasible and scalable supplement to traditional school mental health supports, it is still unknown to what extent this is consistent across different programs, and which individual predictors most consistently promote change in psychosocial wellbeing over time.

School settings are uniquely positioned to provide mental health support, as most youth attend school for approximately 180 days per year while access to standard mental healthcare is often constrained by cost, availability, and workforce shortages (3, 7–10). In turn, mental health support may be particularly needed for early adolescence, a period typically ranging from ages 10–14 that represents a critical period of biological, cognitive, and social development alongside increased vulnerability to anxiety, depression, and attentional difficulties (3, 11). Protective factors during this time span physical and psychological domains, including regular physical activity, perceived competence and self-efficacy, and social connectedness, all of which could be engaged through school-based cycling programs (12–15). From a developmental–ecological perspective, this positions school-based cycling programs as promising multi-purpose interventions, operating directly at the intersection of individual characteristics, social context, and experiential opportunities (1–4, 6).

First, increases in physical activity might be one avenue by which school-based cycling programs can promote psychosocial wellbeing. Physical activity has been associated with improved mood, reduced symptoms of depression and anxiety, and enhanced cognitive and attentional functioning across the lifespan, particularly among individuals with elevated but non-clinical baseline symptoms (11–18). In those with clinical symptom severity, the impact of physical activity-based programs is not yet clear, particularly when implemented as single-component interventions (15). Interestingly, physical activity that occurs outdoors may also benefit from the positive impact on nature on many domains of health. Associations between nature exposure and mood or general wellbeing has been shown in adolescents (19–22), while even viewing nature virtually for as little as six minutes has been shown to have an acute mood-boosting effect (23). Notably, nature exposure alone may not be sufficient to drive meaningful and consistent psychological change across all different types of outdoor environments (24); alongside discrepancies in the types of natural elements accessible in different environments (particularly in urban settings), individual-level factors may determine the degree of impact. For example, nature connectedness is the multi-dimensional trait that represents an individual's affective and psychological attitudes and beliefs towards the natural world (25), and has broader implications for an adolescent's sense of place, self-awareness, social empathy, and relationship skills (26), while in itself being associated with eudaimonic wellbeing (27). Specifically, nature connectedness may influence the degree to which an individual engages with natural elements during outdoor cycling, thus promoting wellbeing in and above passive exposure alone (28, 29). Further, spending more time outdoors may increase nature connectedness longitudinally. Thus, it is important to assess temporal trends in how physical activity, nature exposure, and individual characteristics all play a role to promote wellbeing over time.

Second, self-efficacy has been established as a central part of youth development and mental wellbeing (30). Self-efficacy, both overall and when applied to specific domains such as academics, emotional regulation, or sporting activities, refers to an individual's beliefs in their capabilities to achieve a particular result while coping with potential stressors (31). In physical activity-based interventions, perceived competence has consistently been shown as a key mediator for mental health benefits, with greater gains observed when participants experience mastery in the activity and receive feedback on their performance (32). Structured school cycling programs provide adolescents with repeated opportunities to build self-efficacy related to bicycling, incorporating explicit instruction, behavioral rehearsal, and evaluative tasks, engaging in progressive skill challenges such as balancing, bike control, scanning, and group riding all within a supportive instructional context.

Third, social connectedness is a multi-dimensional construct referring to the degree to which individuals feel safe, supported, and respected in relationships with peers, adults, and the broader community, while encompassing cognitive, psychological, and behavioral dimensions (33). Strong peer support can serve as a protective factor for mental wellbeing in early adolescence, a time where youth begin to spend more time with peers rather than parents (34, 35). Group-based cycling programs naturally facilitate peer interaction, mentorship, and a sense of belonging, which are recognized as mechanisms linking physical activity to mental health benefits (11, 14, 16, 36). Qualitative findings from Brown and colleagues (2) showed that participants perceived improvements in connection to their surroundings following a school-based adventure therapy mountain bike program, alongside increases in focus, competence, and overall physical and mental wellbeing that could potentially act as a buffer during periods of heightened social disruption such as the COVID-19 pandemic. Beyond interpersonal relationships, adolescents' subjective perceptions of their social environments appear to play a critical role in mental health. Muller et al. (37), suggest that perceived neighborhood safety, cohesion, and support are more strongly associated with emotional and behavioral outcomes than objective environmental indicators such as greenspace availability or area-level deprivation. This supports the use of subjective self-report measures as meaningful gauges of wellbeing, and suggests that cycling programs may influence mental health by reshaping adolescents' experiential relationship with their surroundings through repeated, supported engagement in outdoor spaces.

Cycling programs intersect all the forementioned aspects of psychosocial wellbeing, while also remaining enjoyable and accessible to many people. Reports indicate that cycling is one of the most popular forms of outdoor activity in the United States, with an estimated 17.2% of individuals above the age of six (38) and 35% above the age of three participating at least once yearly (39). When focusing specifically within youth, bicycle engagement has increased in recent years, from 49% to 56% among ages three to 17. Importantly, cycling tends to be a naturally enjoyable activity for this age range, which can support positive developmental outcomes. Fromel and colleagues (40) posited that participating in preferred vs. unpreferred activities (and particularly if those activities occur outdoors) is associated with higher wellbeing in adolescents, and a greater likelihood for meeting physical activity recommendations. This highlights the strong momentum in the sport and the nigh opportunity for community and school partnerships to facilitate access to cycling in youth, providing equipment, social groups, and safe cycling instruction that can boost participation and positive outcomes.

Despite the clear potential of such programs, there has been limited research assessing the impact of youth cycling programs on mental wellbeing. A small handful of studies have examined the effects of mountain biking on youth wellbeing, with a recent scoping review by Zarbiv et al. (41) identifying only five studies meeting inclusion criteria and underscoring the limited evidence base in this area. Across these five studies, mountain biking was consistently associated with improvements in self-esteem, resilience, emotional regulation, and social connectedness. Authors identified that key facilitators of positive outcomes were mentorship, peer support, and engagement with natural environments, while these studies were subject to methodological limitations such as reliance on self-report measures, limited use of randomized or controlled designs with a lack of non-riding comparison groups, and insufficient attention to long-term outcomes. In other evaluations of community-mentored mountain biking programs, adolescents have reported increased self-esteem, reduced depressive symptoms, and enhanced social connectedness (36, 42). Whittington and colleagues (42) facilitated a 5-day program for adolescent girls (*n* = 87) ranging from 10 to 16 years of age and from diverse backgrounds ranging from foster care, homelessness, abuse survivors, to well-resourced families. Their findings yielded significant improvement across resiliency domains that were maintained at one-month post program. Despite girls from higher social-economic (SES) backgrounds starting with higher baseline resilience, both low- and high-SES participants showed equivalent improvements, therefore the program was equally effective regardless of economic background.

In addition to mountain biking, two large-scale evaluations have been conducted on school-based cycling programs. Dementyev et al. (4), examined participation in a middle school cycling program across 20 North American schools during the COVID-19 pandemic using a pre–post design. Their results yielded small but statistically significant improvements in wellbeing and reductions in internalizing, externalizing, and attention-related symptoms following program participation, while highlighting differential effects observed by gender, race/ethnicity, and socioeconomic status. Further, Murillo et al. (6), expanded this work across 31 U.S. middle schools and observed modest increases in wellbeing, even alongside small increases in psychosocial symptom scores. Initial regression analyses identified strong associations between wellbeing and modifiable behavioral factors such as sleep duration, screen time, and physical activity levels when examined independently. Together, these findings suggest that school cycling programs may produce population-level shifts in wellbeing, while also highlighting the influence of contextual and behavioral moderators. The generalizability of these findings across different in-school or after-school programs, with variations in frequency, duration, and type of cycling (mountain bike vs. road cycling, for example), is unknown.

Finally, some studies embedded biking within broader experiential, therapeutic, or mentoring contexts. For example, one study by Brown et al. (3), employed a randomized comparative design to examine the differential effects of a school-based mountain biking program with and without integrated adventure therapy components. Middle school students were assigned to either an adventure therapy-integrated cycling group or a cycling-only group. Resilience was assessed across multiple time points, including a one-month follow-up. Students in the adventure therapy group demonstrated sustained increases in resilience, whereas those in the cycling-only group showed more variable and transient improvements. These findings suggest that while cycling may confer physical and social benefits, intentional therapeutic processes may be necessary to support durable psychosocial impact, making it difficult to isolate cycling as an independent mechanism of change.

Taken together, existing evidence suggests that cycling programs may be uniquely positioned to impact multiple facets of mental wellbeing, while remaining feasible to implement in school-based settings. However, important gaps remain: first and foremost, a lack of within-subject longitudinal design precludes the ability to assess individual-level changes in psychosocial wellbeing over time. Secondly, it is unclear which contributing factors have the greatest relative impact, such as baseline mental health, demographic characteristics, physical activity, skill development and self-efficacy, nature exposure, and social connectedness, as most studies have focused on one variable in isolation. To address these gaps, we take an exploratory, within-subject, multivariate approach, examining pre-to-post changes and modeling individual-level predictors of wellbeing (and their collinearity) over the course of a school-based, outdoor cycling program with the aim of finding the most informative yet parsimonious model. As part of a program evaluation for an after-school cycling program called “Bike Club”, we sought to answer the following research questions: 1) what effect does an after-school cycling program have on adolescent psychosocial wellbeing when using a within-subject longitudinal design? 2) What combination of behavioral and demographic predictors are associated with wellbeing scores across pre- and post-program assessments? This multi-pronged strategy allows for nuanced exploration of youth wellbeing and the potential impact of cycling programs, to clarify how, for whom, and under what conditions school-based cycling interventions can most effectively support adolescent mental health, while better understanding its multi-faceted nature.

## 2 Materials and methods

### 2.1 Study participants

We studied fourth- through eighth-grade students (ages 8 through 13 at the pre-program timepoint) during their participation in a year-long after-school bicycling program called “Bike Club”. Bike Club occurred across 31 elementary schools and six middle schools in Tulsa, Oklahoma. Students were recruited at the beginning of the school year via on-campus announcements and advertisements led by the on-site faculty sponsor. Of the 556 students who completed a survey at the pre-program timepoint and 477 at the post-program timepoint, we filtered to only those 329 students who had complete psychosocial wellbeing questionnaires from both timepoints, in order to examine within-subject data for the primary outcome variable (Table 1).

**Table 1:** Demographic characteristics (*n* = 318; *n* = 11 are missing demographic data).

Demographic variable	Participant distribution
Grade	4th (25%, *n* = 82)
	5th (66%, *n* = 217)
	6th (4%, *n* = 12)
	7th: (2%, *n* = 8)
Age at Pre-Program Timepoint	8 years (1%, *n* = 2)
	9 years (19%, *n* = 63)
	10 years (56%, *n* = 185)
	11 years (14%, *n* = 46)
	12 years (5%, *n* = 17)
	13 years (0.3%, *n* = 1)
	Missing data (5%, *n* = 15)
Gender	Female: 53% (*n* = 167)
	Male: 47% (*n* = 151)
Race & Ethnicity	Hispanic: 43% (*n* = 131)
	Black or African American: 19% (*n* = 62)
	White: 19% (*n* = 59)
	Multi-racial (2 or more): 13% (*n* = 42)
	American Indian or Alaskan Native: 4% (*n* = 12)
	Asian or Pacific Islander: 4% (*n* = 12)
Economically Disadvantaged	Yes: 79% (*n* = 252)
	No: 21% (*n* = 66)
Special Education Status	Yes: 10% (*n* = 33)
	No: 90% (*n* = 285)
English Language Learner	Yes: 32% (*n* = 103)
	No: 59% (*n* = 187)
	Former ELL but tested out: 1% (*n* = 4)
	Unknown ELL status: 7% (*n* = 23)

### 2.2 After-school cycling program curriculum

For elementary school sites, Bike Club met once per week for the whole school year, while middle school clubs met twice per week for the fall semester only (mean 21 ± 3.6 sessions, range from 16 to 32). Each session was approximately 1.5 h long and followed a progressive curriculum led by Bike Club staff or trained faculty sponsors alongside adult volunteers. The 20-session curriculum included eight lessons of bicycle skill-building and safety (e.g., helmet fit, bicycle safety check, starting and stopping, scanning and signaling, riding in a group) followed by off-campus group adventure rides to visit parks, ice cream shops, fire stations, and other local places in the surrounding community. All curricula are publicly available on Bike Club Tulsa's website (bikeclubtulsa.com). Each session aimed to achieve at least 20 min of moderate-to-vigorous physical activity per session, while also fostering secondary goals such as teamwork, social connections, and building healthy lifestyle habits. The curriculum finished with a capstone experience: for middle school students, this was a day-long field trip to the mountain bike trails for skill-building and trail riding. For elementary school students, this was the “Bike Club Rally”, a day-long field trip with all 31 elementary sites together, riding over four miles from downtown Tulsa to a local park for lunch, ice cream, and outdoor play.

### 2.3 Survey collection

Pre- and post-program surveys were administered on paper during the first and last meetings of the program, respectively. Students were instructed to answer all questions to the best of their ability, were ensured that their responses would remain anonymous, and were given a time limit of thirty minutes. In some cases, adults helped to read the questions aloud to students who reported difficulties with reading. The survey included the following quantitative measures: 1) psychosocial wellbeing via the World Health Organization (WHO-5) Wellbeing Index, using the sum of all five questions (43), 2) health behaviors (including physical activity, outdoor time, and perceived help from adults with emotions) via the U.S. Centers for Disease Control and Prevention's Youth Risk Behavioral Survey (YRBS) (44, 45), 3) connection with nature, taking the median score from via an abridged 5-question version of the Connection with Nature Index (CNI) (25), 4) bike self-efficacy, using the median score from a custom scale, with responses from zero (never) to 10 (always) on eight questions regarding bicycling confidence, safety (helmet use, bike safety check), knowledge (bike parts), and skills (shifting gears, using hand signals, riding in a group). To justify our use of the median score for the latter two scales, we confirmed that all components had positive correlations with each other at both time points (*r*'s = 0.28–0.59 with the mean *r* = 0.39 at pre, and *r*'s = 0.32–0.56 with the mean *r* = 0.45 at post, for connection with nature; *r*'s = −0.11–0.44 with the mean *r* = 0.18 at pre, and *r*'s = −0.05–0.34 with the mean *r* = 0.1 at post for bike self-efficacy).

All the data included in this study were collected for routine program evaluation and quality improvement purposes rather than for research, and this manuscript was prepared using pre-existing, de-identified information. In accordance with institutional policies and applicable federal regulations, this project did not require Institutional Review Board review.

### 2.4 Statistical analysis

All statistical analyses were performed in R. To answer our first research question assessing the overall effect of the after-school cycling program, we examined group-level changes in all survey-derived variables from pre- to post-program. For all continuous variables, we first checked whether pre-to-post differences were normally distributed (using Shapiro–Wilk tests via the “shapiro.test” function in R). If so, we used paired sample *t*-test (“*t*.test” function in R) to test for significant pre-to-post changes, and if not we used the Wilcoxon Signed-Rank test (“wilcox.test” function in R). For all categorical variables, we used ordered logistic regression with paired samples (“polr” function from the *MASS* package in R).

Second, we measured collinearity between all survey-derived variables using pairwise Pearson R correlations, separately for pre- and post-program timepoints. With the exploratory nature of the analysis, this allowed for a preliminary assessment of variable relationships and how these relationships may change over time.

Finally, associations with wellbeing were examined via linear mixed effects regression (“lmer” function from the *lmerTest* package in R) using backwards stepwise variable selection via AIC criterion (“step” function in R). With WHO-5 scores as the outcome variable, the following variables were included as possible predictors: timepoint (i.e., pre- or post-program), self-reported survey variables (days per week of physical activity, outdoor time per day, perceived help with emotions, bike self-efficacy, and connection with nature), and demographic information (gender, ethnicity, economic disadvantage, special education status, and English language learning status), with a random intercept for each participant. A final regression model was created with all variables that were preserved after backwards selection, and effect size was calculated for all statistically significant main effects (“eta_squared” function in R). To interpret significant main effects and their impact on wellbeing, *post-hoc* tests were performed via estimated marginal trends for numeric variables, or estimated marginal means for categorical variables (“emtrends” or “emmeans” functions, respectively, from the *emmeans* package in R). For categorical variables with more than two levels, we did not do pairwise comparisons; instead, to limit the number of comparisons and aid interpretation, all levels were compared to one baseline category (i.e., *“*Never” getting help with emotions, or an ethnicity of “White” compared to other minority groups). Notably, we did not include any formal interaction effects in our regression analysis, in order to reduce model complexity and improve interpretability. Thus, we are unable to model whether or not the associations may change over time– for example, whether physical activity has a stronger association with wellbeing before or after the program, or if certain predictors variables meaningfully interacted with one another to impact wellbeing scores. Additionally, we had an insufficient sample from each of our 37 sites to meaningfully interpret site-specific differences, and due to the retrospective study design we did not measure any other school-level variables that might aid interpretation (such as social climate or staff engagement). To examine the potentially confounding influence of school site, we performed a supplementary exploratory analysis that included school site as an additional random effect in the regression model, with student nested under school while keeping all other fixed effects the same.

## 3 Results

### 3.1 Pre- to post-program scores in survey-based variables in after-school cycling club participants

There was no statistically significant difference in overall group mean psychosocial wellbeing after the cycling program [mean ± SE = 70.4 ± 1.1 and 69.5 ± 1.1 for pre- and post-program, respectively; *t*(328) = 0.77, *p* = 0.44] (Figure 1A). However, over half of students still showed a clinically meaningful change when using 10 points on a 100-point scale as the criteria: 27% of individuals (*n* = 88) increased scores, 29% (*n* = 92) decreased, and the remainder (45%, *n* = 147) did not change. While the minimum clinically important difference is not well-established for this measure, a 10-point change has been shown to correspond to differences in clinical symptom severity, and is used here as a general indicator of non-arbitrary change (43). Furthermore, participants' scores at baseline also influenced the magnitude of change (*β* = −0.62, R^2^ = 0.32, *p* < 0.001), with those reporting lower wellbeing (i.e., 15 or less) at the beginning of the program generally showing positive change while those reporting higher wellbeing (i.e., 20 or more) generally showing negative change.

**Figure 1:**
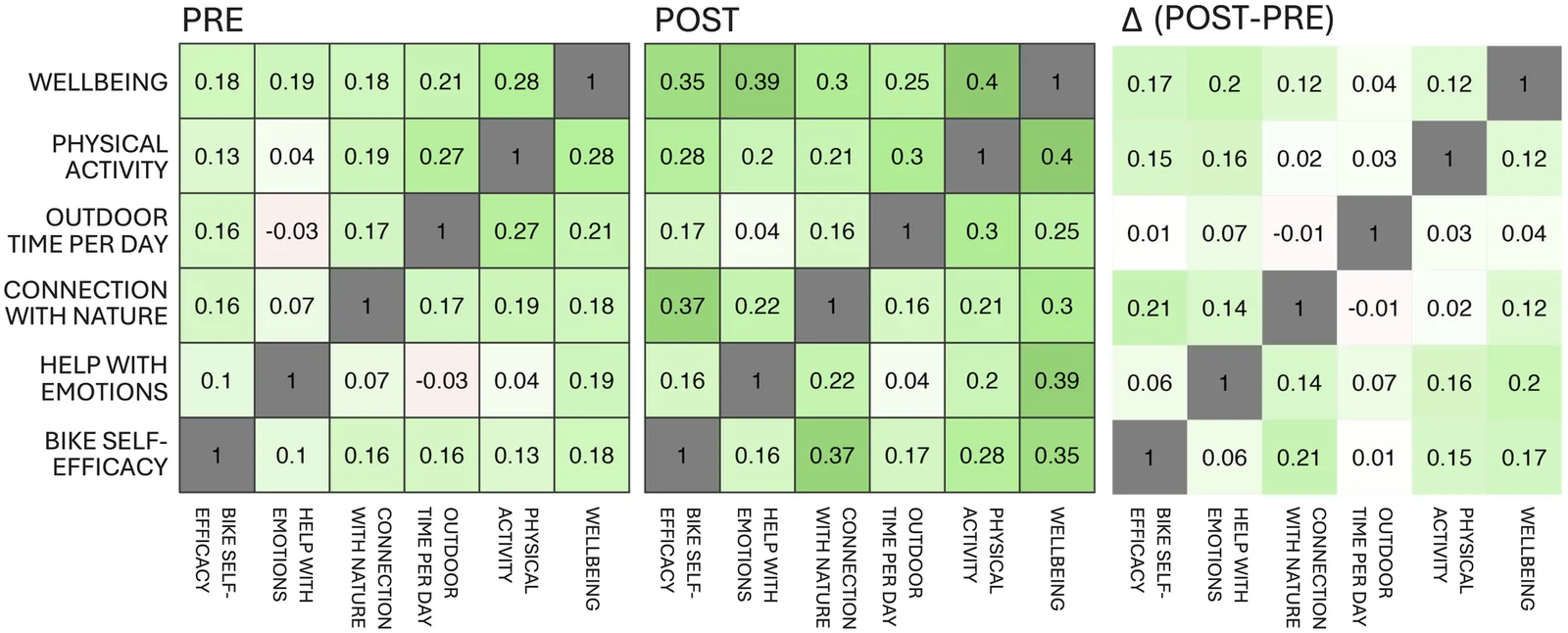
Collinearity of survey-derived variables. All correlations are Pearson R values, uncorrected, showing the pre-program timepoint (left panel), post-program timepoint (middle panel), and the change in correlation across timepoints (right panel).

In addition, there were no significant differences in days per week of physical activity (mean ± SD = 4.9 ± 2.1–5.1 ± 2.0, *t*_321_ = −1.1, *p* = 0.27) (Figure 1B) or perceived help with emotions (*β* = −0.21, *χ*^2^ = 1.58, *p* = 0.21) (Figure 1E). However, bike self-efficacy (mean ± SD = 5.1 ± 4.0–9.3 ± 1.4, V = 1,456, *p* < 0.001) (Figure 1D) and outdoor time per day (*β* = −0.81, χ^2^ = 22.8, *p* < 0.001) (Figure 1F) significantly increased, while connection with nature significantly decreased (mean ± SD = 9.1 ± 1.9–8.5 ± 2.4, V = 8,143.5, *p* < 0.001) (Figure 1C).

### 3.2 Collinearity of survey-derived variables

All predictor variables that were included in the model building had weak to moderate correlations with each other at the pre-program (*r*'s = −0.03–0.28, mean *r* = 0.15) and post-program (*r*'s = 0.04–0.40, mean *r* = 0.25) timepoints (Figure 2). Narrowing focus to how these variables were associated with psychosocial wellbeing, all had small to moderate correlations with WHO-5 scores at both timepoints (*r*'s = 0.18–0.28 at pre; 0.25–0.40 at post).

**Figure 2:**
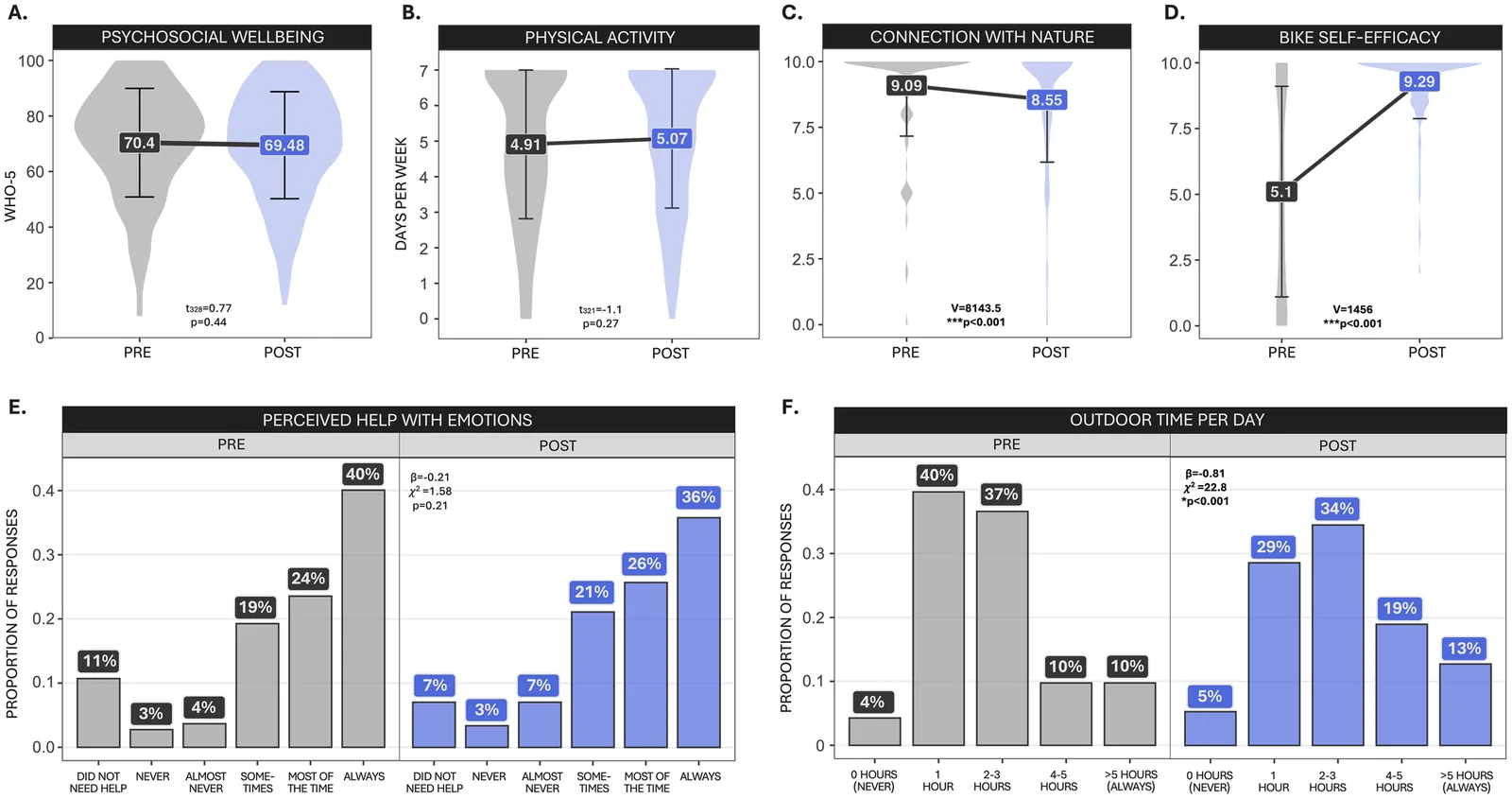
Effect of an after-school cycling program on survey-derived self-report variables at the pre- and post-program timepoints, including (**A**) psychosocial wellbeing, (**B**) physical activity, (**C**) connection with nature, (**D**) bike self-efficacy, (**E**) perceived help with emotions, and (**F**) outdoor time per day.

### 3.3 Factors associated with psychosocial wellbeing while participating in an after-school cycling club

After backwards stepwise variable selection, the final mixed effects regression model (R^2^ = 0.50, AIC = 3,166) included eight variables significantly related to psychosocial wellbeing: perceived help with emotions, days per week of physical activity, connection with nature, outdoor time per day, bike self-efficacy, gender, ethnicity, and English language learner status, as well as a random intercept for each participant. Based on this, the following formula was used for the final mixed-effects regression model: WHO-5 score ∼ days per week of physical activity + perceived help with emotions + outdoor time per day + connection with nature score + bike self-efficacy score + gender + ethnicity + English language learner status + (1|student). All significant main effects and *post-hoc* comparisons are summarized in Table 2. Special education status [F(285) = 0.02,*p* = 0.88], economic disadvantage [F(283) = 0.5, *p* = 0.47], and timepoint [F(387) = 3.4, *p* = 0.07] were eliminated from the model. Sensitivity analysis that included student nested under school as an additional random effect produced nearly identical results (R^2^ = 0.50, AIC = 3,151) (Supplementary Table S1).

**Table 2:** Determinants of psychosocial wellbeing via mixed effects linear regression with backwards stepwise variable selection.

Fixed Effect	*F* (df)	*p*	Effect size (eta-squared; magnitude)	Post-hoc comparison (estimated marginal mean/trend ± SE)
Perceived help with emotions	12.0 (537)	<0.0001	0.10 (medium)	Never: 14.3 ± 1.2
				Almost never: 14.1 ± 0.8
				Sometimes: 15.7 ± 0.5
				**Most of the time: 17.8** **±** 0.5* [*t*(543) **=** **3.0, *p*** **=** **0.003]**
				**Always got help:18.4** **±** 0.4* [*t*(548) **=** **3.5, *p* < 0.001]**
				Did not need help: 16.8 ± 0.6
				*significantly different than “Never” as a baseline comparison
Physical activity (days per week, self-reported)	35.2 (547)	<0.0001	0.06 (medium)	Trend: **0.54** **±** 0.1* [*t*(566) **=** **5.8, *p* < 0.001]**
English language learning status	16.1 (283)	<0.0001	0.05 (small)	No: 14.9 ± 0.4
				**Yes: 17.5** **±** 0.6* [*t*(305) = −3.92, *p* **=** **0.0001]**
Ethnicity	2.8 (282)	0.02	0.05 (small)	White: 16.8 ± 0.6
				Black/African-American: 17.8 ± 0.6
				Hispanic: 16.3 ± 0.4
				Multi-racial: 16.7 ± 0.7
				American Indian or Alaska Native: 16.1 ± 1.1
				**Asian or Pacific Islander: 13.3** **±** 1.1* [*t*(303) = −2.8, *p* **=** **0.006]**
				*significantly different than “White” as a comparison
Gender	13.4 (288)	0.0003	0.04 (small)	Male: 16.9 ± 0.4
				**Female: 15.5** **±** 0.5* [*t*(312) = −3.6, *p* **<** **0.001]**
Connection with Nature	10.5 (559)	0.001	0.02 (small)	Trend: **0.27** **±** 0.09* [*t*(579) **=** **3.2, *p*** **=** **0.001]**
Outdoor time per day	2.5 (552)	0.04	0.02 (small)	0 h (never outside): 14.6 ± 0.9
				1 h: 16.0 ± 0.4
				2–3 h: 16.2 ± 0.5
				**4–5 h: 17.0** **±** 0.6* [*t*(560) **=** **2.6, *p*** **=** **0.01]**
				**<5 h: 17.2** **±** 0.6* [*t*(576) **=** **2.6, *p*** **=** **0.01]**
				*significantly different than “0 h” as a baseline comparison
Bike self-efficacy	5.8 (432)	0.02	0.01 (small)	Trend: **0.11** **±** 0.05* [*t*(449) **=** **2.4, *p*** **=** **0.02]**

Bold text indicates significant difference in the estimated marginal trend, or the estimated marginal mean compared to the reference category.

## 4 Discussion

Psychosocial wellbeing did not significantly change at the group level from pre- to post-program, however substantial individual variability in wellbeing scores was observed. Stepwise regression revealed that multiple lifestyle, personal, and demographic factors were associated with wellbeing scores, with perceived social support and self-reported physical activity having the largest relative impacts alongside bike-related self-efficacy, connection with nature, daily outdoor time, gender, ethnicity, and English language learning status. Bike-related self-efficacy and daily outdoor time were significantly greater at the end of the cycling program compared to the beginning, connection with nature was significantly lower, while the rest showed no significant changes between the two timepoints. Overall, these findings may suggest that while school-based cycling programs can provide positive mastery experiences for youth and may increase time spent outdoors, more intentional program design and facilitation might be needed for these programs to serve as a direct and consistent form of school-based mental health support.

### 4.1 Impact of school-based cycling programs on psychosocial wellbeing

WHO-5 psychosocial wellbeing scores remained relatively stable at the group level from pre- to post-program. (Figure 1). This differs from previous research reporting small but statistically significant improvements in wellbeing following participation in a school-based cycling program (4, 6, 46). This discrepancy could be due to multiple factors—first, as a whole, our sample had slightly above-average wellbeing when compared to normative data for healthy populations (43), which could represent a reduced capacity for improvement. In our sample, students who started the year with lower wellbeing (i.e., 15 or below) generally tended to show an increase in scores, while students who started the year with higher wellbeing (i.e., 20 or above) generally tended to decrease. This could be a limitation of using continuous WHO-5 scores, especially when depending on them as the sole measure for mental wellbeing. Future approaches could incorporate multiple timepoints of measurement, add other specific subscales of wellbeing (such as affective or social), and/or use WHO-5 only as a general screening tool, using the well-established clinical threshold of 50 to better identify high-need students for further intervention (43).

The null finding for WHO-5 could also be due to other program factors such as dosage or frequency, which differ when compared to a similar school-based cycling program called Riding for Focus (R4F) that has shown small but statistically significant improvements in WHO-5 scores across multiple studies (4, 6). While Bike Club offered greater total contact hours than R4F (upwards of 30 vs. 18 h, respectively), these sessions were more spread out across the school year compared to the more intensive 6-week, thrice-weekly R4F program that was based in physical education class. However, we also cannot rule out seasonal effects in the current study, with measurements occurring at the beginning vs. end of the semester. Future research should incorporate a greater number of timepoints (including follow-ups), as well as a non-cycling comparison group, to better delineate the time course of yearly vs. cycling-based wellbeing changes.

Additionally, the age of our participants could impact the findings. While Bike Club occurred primarily for elementary students (over 90% of participants were in 4th or 5th grade), R4F occurred only for middle school. The inadequate sample size of middle school students in the current study precludes the examination of age effects, but given the differences in the school environment between elementary and middle school and the complex interplay of lifestyle factors during the greater transitional period of the latter, it is unclear if middle school students may respond different to cycling programs or be more sensitive to program-derived psychosocial wellbeing changes.

Finally, there are important differences in our statistical approach compared to the R4F studies. The larger sample size in the R4F studies increases the likelihood for statistical significance, while the lack of paired responses precludes the ability to examine individual-level changes that may occur. In the current study, paired data revealed that nearly half of individuals do show substantial increases or decreases in wellbeing that negated each other when calculating the group mean, suggesting that group-level approaches may not always reflect meaningful individual changes. Given the complex nature of mental health and the high degree of inter-individual variability, an individual-level approach may be more appropriate, aggregating within-case changes such as a growth curve analysis or a single-case research design (47).

Overall, this suggests that cycling participation alone might represent just one singular behavioral intervention, but might not be sufficient to translate into broader lifestyle change to generate measurable and significant increases in overall wellbeing or many of its predictors. A more frequent, comprehensive, or intentional approach may be required. One such approach is found in a growing body of adventure therapy research, showing that integration of kinesthetic activities within a therapeutic framework is associated with improvements in resilience, emotional regulation, and broader psychosocial functioning among adolescents, rather than relying on incidental exposure alone (2, 3, 9, 47–49). Core components of a therapeutic framework include challenge-by-choice activities paired with intentional reflection, structured debriefing, and group processing, all which serve to facilitate meaning-making around effort, failure, and mastery. The unique aspects of cycling programs position them well to seamlessly integrate with adventure therapy processes, including technical skill challenges, the social aspects of group riding, experience of variable emotional states alongside access to adult mentors, and nature exposure, which can serve as a key co-facilitator of therapeutic change (3).

### 4.2 Correlates of psychosocial wellbeing in youth participants

Although our initial program evaluation found no significant group-level changes in wellbeing across the cycling program, the use of regression provides an insightful “report card” on self-reported wellbeing in youth participants, showing the multi-faceted nature of mental health and identifying multiple behavioral and demographic variables that may contribute.

First, perceived help with emotions (specifically from adults) had the largest relative association with psychosocial wellbeing, with a medium effect size. This suggests that availability of emotional support is a central component of youth wellbeing at any given time. Although this variable was not significantly altered over the course of the program, cycling programs may offer a way to maintain youth-adult connections over time. Notably, perceived help with emotions is complex and constructed from multiple subcomponents, each of which play a role: one's ability to identify and regulate emotions, one's propensity for help-seeking behavior, and the availability of trusted adults or healthcare providers from which to receive help (50). This reinforces the importance for all school-based programs, whether focused on cycling or other activities, to encourage emotional intelligence and build supportive youth-adult relationships throughout the curriculum.

From a wider perspective, this could be thought of as a specific form of perceived social support, which is defined as the perception that one is cared for and valued within a mutually supportive social network (51). Perceived social support has been shown to correlate with psychological wellbeing in youth, across all socioeconomic levels (52). In turn, nurturing relationships can serve as protective factor throughout youth development, promoting health across psychological, physiological, and neurobiological domains (53, 54). With this in mind, it is unclear if the positive relationship with wellbeing in the current study is specific to social support from adults, or whether it generalizes to perceived social support from peers or to other measures examining the degree of social connectedness. Furthermore, it is unclear how individual vs. group cycling could have similar (or proportionally reduced) impact on wellbeing, or how cycling may differ from other sports or activities that also involve peers and adult mentors (for example, team sports). It may also be that group dynamics must be intentionally leveraged during group physical activity to best promote physical and psychosocial health outcomes (55).

Second, self-reported physical activity was moderately, positively associated with psychosocial wellbeing. This corroborates previous findings across cross-cultural studies showing a consistent correlation between increased physical activity levels and increased psychosocial wellbeing scores (56) as well as reduced prevalence of mental health disorders (57). These relationships may be causal in nature, as a single session of exercise has been shown to benefit the brain through parallel hormonal, microbial, neural (for example, by increasing vagal activity), and psychological mechanisms (58). It is not year clear how this relationship is specific to cycling, or if other team sports, individual sports, or forms of aerobic or anaerobic exercise could elicit similar effects. No matter the modality, continued participation is important for long-term benefits, and cycling has the potential to address the intrapersonal, interpersonal, and structural concerns that contribute to dropout rates in youth sports (59). Future research is needed to better understand the acute and long-term effects of cycling participation on wellbeing, while probing the underlying mechanisms and examining the degree to which they are specific to cycling.

Third, a handful of other survey-derived variables had small but statistically significant associations with WHO-5 scores. Bike-related self-efficacy was positively related to wellbeing while also showing substantial pre- to post-program improvements, suggesting that this domain-specific variable may be a pliant avenue by which cycling programs can promote mental health. Indeed, group cycling touches on all aspects of self-efficacy, including personal and vicarious experiences of mastery, verbal encouragement from others, and navigating various physical and emotional states that are probed from the kinesthetic properties of cycling, all while promoting confident and continued participation in the sport (60). As the increases in this variable were not sufficient to promote changes in WHO-5 scores overall, this speaks towards the multifactorial nature of wellbeing; programs may aim to promote simultaneous changes in multiple predictors at once for the greatest impact. Notably, this self-efficacy measurement also showed a ceiling effect at the post-program timepoint, with responses heavily clustered (i.e., 92% of responses) around the maximum value of 10. This reduces its utility and precludes the examination of any interaction effect across time. Future studies could employ a different type of scale to aid interpretability in regression.

In addition to self-efficacy, both connection with nature and outdoor time per day also positively contributed to psychosocial wellbeing, suggesting that aspects of outdoor experiences are non-negligible components of cycling programs. Future research could incorporate an indoor cycling comparison group to better understand the specific effects of nature exposure. Additionally, these two variables also showed significant changes after the program, with outdoor time per day significantly increasing (with a greater percentage of youth reporting four or more hours outdoors per day, which could be due in part to the hour outdoors during the program itself) and connection with nature slightly decreasing. As the latter is also measured on a 0–10 scale, this could also be due to a ceiling effect, with most students responding very highly on this scale at both timepoints (and many reporting the maximum value of 10). While the utility this type of scale may be limited, this suggests that most young adolescents demonstrate an inherent enjoyment and comfort towards nature and spending time outdoors. To capitalize on this baseline relationship, cycling program facilitators could test the addition of strategic frameworks that intentionally promote connection with nature as an educational outcome, such as the EcoWellness framework (61).

Finally, certain demographic variables also impacted youth wellbeing. Female students showed lower scores than their male counterparts, which has also been found in previous studies (62, 63). Asian-Pacific Islander students were the only racial and ethnic minority group showing lower scores than White students. Notably, this particular group had the fewest students (only 4% of the total sample), so it is unknown if this statistical difference in wellbeing scores would persist in a larger sample. Racial and ethnic minority groups like Hispanics and African-Americans reported comparable wellbeing to White students, which has been previously established—researchers have cited protective factors for minority groups such as family support, religiosity, and ethnic diversity in friendships (64). Finally, monolingual native English speakers showed lower wellbeing scores than English language learners (ELL), which links with previous findings examining social-emotional wellbeing (65). While lack of English proficiency can be a stressor for youth, it could be related to increased resiliency through adaptation to stress, along with cultural protective factors (66). In order to best promote mental health at the individual level, these findings suggest that cycling program facilitators should be aware of how demographic subgroup variability may impact wellbeing, while future research could examine the intersectionality of these factors and the unique protective factors at play for specific aspects of wellbeing (67).

When examining all variables together, findings from our regression analysis paint a multi-faceted picture of wellbeing: perceived social support from adults, physical activity, self-efficacy, time outdoors, and connection with nature are all adaptable factors that contribute to wellbeing, while participant demographics such as gender, race and ethnicity, and language status also play a role. Although only self-efficacy scores and daily outdoor time were statistically different from pre- to post-program, from an anecdotal perspective it is likely that all variables were engaged to some degree throughout the program, given its design. Taking into account the multi-faceted nature of wellbeing, it is perhaps a strength of cycling programs to cast a wide net, allowing each participant to connect with aspects of cycling that feel most health-promoting to them. Future studies could design cycling interventions that intentionally promote each of these elements, testing the feasibility of cycling programs to serve as school-based mental health supports.

### 4.3 Limitations

While our study offers insight on the psychosocial impact of a youth cycling program, several limitations are present, including features of the program and study design that constrain interpretation of our findings. First, as a single-group program evaluation, we did not include a non-cycling comparison group. Thus, observed differences in pre-post timepoints cannot be specifically attributed to the after-school bike program, and in turn, the absence of change in wellbeing outcomes should not be interpreted as definitive evidence that the program had no effect. Confounding and unmeasured variables such as developmental maturation, seasonal changes, academic stressors, changing group dynamics or social contexts, etc., may have contributed to participants' scores across the program. Similarly, with regard to survey administration, having only two timepoints of data collection with no long-term follow-up assessments limits the ability to model the trajectory of wellbeing change over the course of the program and beyond, to identify sensitive periods for intervention or to explore whether certain phases of the program (i.e., skill-building lessons vs. neighborhood adventure rides) may differentially impact wellbeing. Future studies would benefit from adding additional timepoints for data collection, such as program midpoints as well as follow-ups.

Secondly, our dependence on self-report questionnaires by youth participants highlights potential response bias. For example, all of the survey-derived self-report variables significantly contributed to wellbeing in our regression model; while each survey tool was based on independently-validated instruments (i.e., WHO-5, YRBS) with established psychometric properties, research indicates that self-report data, especially among youth, may be susceptible to acquiescent responding, social desirability, or a generalized tendency to endorse higher or lower values across questions irrespective of the content (68, 69). Future research would benefit from complementing self-report measures with objective assessments, such as accelerometer-derived physical activity metrics, ecological momentary assessment of mood or affect, psychophysiological tasks, biomarkers of stress and wellbeing, or teacher- and parent-reported measures of social-emotional and behavioral health, as well as a mixed-methods approaches that incorporate qualitative analyses. This would also serve to improve the validity of wellbeing assessments by allowing for cross-validation of self-reported outcomes.

Thirdly, we did not model differences across school sites, despite their potential impact on program outcomes. Future studies could benefit from the collection of site-specific variables that might effect students' experiences and the resultant impact on wellbeing, such as school climate, teacher or parental engagement, or aspects of the physical environment in the surrounding neighborhood (access to parks, greenspace, or cycling infrastructure).

Finally, in terms of generalizability of findings, our sample was predominantly composed of fourth- and fifth-grade elementary school students (over 90% of participants), with a limited representation of middle school students. This occurred due to program structure rather than research design, and while it provides useful insight into the effects of cycling and determinants of wellbeing in late childhood, it limits comparability with previous school-based cycling studies focusing on a middle school age group. As suggested by these previous studies, the transitional period of early adolescence characterized by heightened social sensitivity, increased peer influence, and elevated vulnerability to internalizing symptoms might be more pronounced in this older group, and thus may represent a more effective window for pronounced or durable psychosocial benefits from cycling program participation (2, 3, 9, 10, 48).

## 5 Conclusion

A year-long, once-weekly after school cycling program did not consistently impact self-reported psychosocial wellbeing at the group level, although two-thirds of individuals still showed clinically meaningful changes across time. The use of stepwise regression analysis showcased the multi-faceted nature of wellbeing, with perceived social support and self-reported physical activity having the strongest associations with WHO-5 scores, while cycling self-efficacy, connection with nature, time spent outdoors, and demographic variables like gender, race and ethnicity, and English language learner status also had positive associations.

More broadly, these results have implications for the facilitation of physical activity-based youth programs, with benefits stretching far beyond the kinesthetic activity itself. Framed as positive youth development interventions, these programs have the potential to influence modifiable and interrelated correlates of psychosocial wellbeing; however, incidental exposure alone may not be sufficient to drive consistent change and to serve as a school-based mental health support. Future work can examine how cycling programs can be intentionally structured and facilitated to yield more consistent psychosocial benefits, amplifying youth mental health outcomes while accounting for the complex and individualized nature of wellbeing.

## Data Availability

The data analyzed in this study is available upon request. Requests to access these datasets should be directed to emily@humblesons.org.
