# Supplementary material for: Determinants of psychosocial wellbeing within a year-long youth cycling program
Source: Front Sports Act Living. 2026 Oct 19;8:1892082. doi: 10.3389/fspor.2026.1892082 (PMC13725881; doi:10.3389/fspor.2026.1892082)
Supplement: Supplementary file 1 [file Table1.docx]

**Supplementary Table 1.** Determinants of psychosocial wellbeing via mixed effects linear regression with backwards stepwise variable selection, when including student nested under school site as an additional random effect. After variable selection, the resulting formula is as follows: WHO-5 score ~ days per week of physical activity + perceived help with emotions + outdoor time per day + connection with nature score + bike self-efficacy score + gender + ethnicity + English language learner status + (1 | school/student). The mean absolute change in F-statistics of 14% compared to the original model. The difference of effect size estimates is 0.01 or no change. The only difference in post-hoc comparisons is one additional contrast showing statistical significance for perceived help with emotions, where students who reported they “did not need help” had higher wellbeing versus those who “Never” received help.

| **Fixed Effect** | ***F*(df)**  **(% change from previous model)** | ***p*** | **Effect Size** | **Post-hoc comparison** |
| --- | --- | --- | --- | --- |
| **Perceived help with emotions** | 12.7 (539)  (+5.8%) | <0.0001 | 0.11 (medium)  (+0.01) | Never: 14.3±1.2  Almost never: 14.1±0.8  Sometimes: 15.8±0.5  **Most of the time: 17.8±0.5* (t(547)=3.0, p=0.003)**  **Always got help:18.5±0.4* (t(552)=3.7, p<0.001)**  **Did not need help: 17.0±0.7* (t(545)=2.2,p=0.03)**  **significantly different than “Never” as a baseline comparison* |
| **Physical activity (days per week, self-reported)** | 43.0 (550)  (+22%) | <0.0001 | 0.07 (medium)  (+0.01) | Trend: **0.59±0.09***  **(t(568)=6.40, p<0.0001)** |
| **English language learning status** | 18.0 (267)  (+12%) | <0.0001 | 0.06 (medium)  (+0.01) | No: 14.9±0.45  **Yes: 17.6±0.62***  **(t(292)=-4.13, p<0.0001)** |
| **Ethnicity** | 3.7 (273)  (+32%) | 0.003 | 0.06 (medium)  (+0.01) | White: 16.8±0.6  Black/African-American: 18.0±0.7  Hispanic: 16.3±0.5  Multi-racial: 16.7±0.7  American Indian or Alaska Native: 16.3±1.0  **Asian or Pacific Islander: 13.1±1.1* (t(303)=-3.1, p=0.002)**  **significantly different than “White” as a comparison* |
| **Gender** | 15.9 (289)  (+18.7%) | <0.0001 | 0.05 (small)  (+0.01) | Male: 17.0±0.48  **Female: 15.5±0.49***  **(t(315)=-3.86, p=0.0001)** |
| **Outdoor time per day** | 3.2 (550)  (+28%) | 0.01 | 0.02 (small)  (no change) | 0 hours (never outside): 14.6±0.9  1 hour: 16.0±0.5  2-3 hours: 16.2±0.5  **4-5 hours: 17.0±0.6* (t(562)=2.7, p=0.008)**  **<5 hours: 17.2±0.7* (t(573)=2.9, p=0.003)**  **significantly different than “0 hours” as a baseline comparison* |
| **Connection with nature** | 8.5 (554)  (-19%) | 0.004 | 0.02 (small)  (no change) | Trend: **0.24±0.08***  **(t(574)=2.85, p=0.005)** |
| **Bike self-efficacy** | 5.0 (431)  (-14%) | 0.03 | 0.01 (small)  (no change) | Trend: **0.10±0.05***  **(t(450)=2.19, p=0.03)** |

***Bold text indicates significant difference in the estimated marginal trend, or the estimated marginal mean compared to the reference category.**
